# Microbiological Quality of Australian Beef, Sheep and Pork Carcases, Cuts and Offals

**DOI:** 10.3390/foods12203832

**Published:** 2023-10-19

**Authors:** Jessica Jolley, Andreas Kiermeier, John Sumner

**Affiliations:** 1 South Australian Research and Development Institute, Adelaide, SA 5064, Australia; 2 Statistical Process Improvement Consulting and Training Pty Ltd., Gumeracha, SA 5233, Australia; andreas.kiermeier@gmail.com; 3 M&S Food Consultants Pty Ltd., Deviot, TAS 7275, Australia; john_sumner2@bigpond.com

**Keywords:** microbiological quality, beef, sheep, pig, carcases, cuts, offals

## Abstract

A one-year survey was undertaken of the microbiological quality of carcases and the derived primal cuts, manufacturing meat and offals at twelve Australian export establishments (six beef, three sheep/lamb and three pork). A total of 27,157 microbiological results for aerobic plate count (APC) and generic *Escherichia coli* were gathered, 15,155 from beef, 8405 from sheep and 3597 from pig establishments. The mean log_10_ APCs on beef, sheep and pig carcases were 0.84, 1.60 and 1.30 log_10_ cfu/cm^2^, respectively. For primals, the mean log_10_ APC was higher for beef but was similar for sheep and pork primals, with ‘outside’ cuts having higher counts. For manufacturing meat, the concentration was 2–3 log_10_ cfu/g, irrespective of species. The prevalence (%) of generic *E. coli* from beef, sheep and pork was 2.3, 28.4 and 5.4 on carcases; 7.0, 20.6 and 3.2 on primals; and 5.8, 33.6 and 6.1 on manufacturing meat, respectively. The mean log_10_ APCs of beef, sheep and pork offal were 3.23, 3.18 and 3.37 log_10_ cfu/g, with tripes and tongues having APCs 1–2 log_10_ units higher than organ offals. The results reflect improvements in total bacterial loadings compared with previous national baseline surveys.

## 1. Introduction

Following several food poisoning incidents associated with the consumption of hamburgers, the Food Safety and Inspection Service in the United States introduced the *Pathogen reduction: hazard analysis and critical control point (HACCP) systems; final rule*, also known as the ‘Mega Reg’ [1]. As a major exporter to the USA of manufacturing meat for grinding, in 1998, Australia mandated a government-supervised monitoring program for carcases, the *E. coli* and *Salmonella* Monitoring (ESAM) program. The ESAM program is performed by all export establishments, which are required to respond to results considered unacceptable based on a three-class sampling plan and a moving window [2], the original criteria having been set using 2001 data [3]. The results are stored in a national database which is “active”, with each export establishment being able to generate reports and summaries of their data and the national microbiological profile.

In the 25 years since the inception of mandatory monitoring, the Australian industry has undergone significant improvements in infrastructure and in process control. These changes were documented by a series of national baseline studies of beef and sheep carcases and cuts with a trend towards improved microbiological profiles of both categories [4,5,6,7,8,9,10,11]. Typically, few samples, particularly beef, had *E. coli* counts above the limit of detection, prompting establishments to question the utility of *E. coli* testing of carcases as it provided no meaningful relationship with end-product verification testing or port-of-entry testing.

This thinking, together with a parallel trend over the same period of a decrease in marketing of carcases per se and of increased processing of meat cuts and offals, led to a review of the microbiological monitoring of Australian meat [12]. The review, undertaken with representatives from industry and the controlling authority (the Department of Agriculture, Fisheries and Forestry, DAFF), canvassed the microbiological monitoring regimes of other meat exporting countries, analysed the ESAM database and recommended an industry trial be undertaken to provide baseline data on carcases, primals (individually packed), manufacturing or bulk-packed meat and offals. Accordingly, the trial was undertaken at six beef, three sheep and three pig establishments and generated more than 20,000 data points for carcases, primals, bulk meat and offal [13]. The resulting database provides a unique linkage between the carcase and products derived from it, bulk meat, primals and offals, and is described in the present paper. In addition, it was the intention to use these data to develop alternative microbiological criteria by which to assess the performance of the Australian meat industry and to submit them for review by Australia’s major trading partners.

## 2. Materials and Methods

### 2.1. Selection of Establishments

Twelve establishments (six beef, three sheep and three pig) were selected from the Australian states of Queensland, New South Wales, Victoria, South Australia, Western Australia and Tasmania. An additional selection criterion was based on the size of the establishment and, hence, slaughter volume; other process characteristics of each establishment are presented in Table 1, Table 2 and Table 3.

As indicated in Table 1, the slaughter volume/hour varied considerably across the six participating beef establishments, with two of the larger establishments, C and D, having separate hide-on and hide-off areas and a hot water intervention followed by spray chilling. A two-knife cleaning system, where one knife resides in an 82 °C water bath while the other is in use, was employed by beef establishments exporting to the European Union.

Sealing of the bung with a plastic bag and elastic band was standard practice in all beef and sheep establishments, except for establishment I (Table 2) and all three pig processors (Table 3). All three sheep establishments used steam/vacuum devices to remove macro contamination on cutting lines and sheep establishments G and I operated a two-knife system.

As indicated in Table 3, pig slaughter volumes/hour varied considerably and one processor (K) used steaming to loosen the bristles, an operation considered superior to water scalding where build-up of organic material occurs in the scald tank [14].

### 2.2. Sampling Regime

All samples were collected over a 13-month period from October 2017 to October 2018. Carcases and bulk meat were sampled after overnight chilling according to the *Microbiological Manual for Sampling and Testing of Export Meat and Meat Products* [2] at a frequency of 1 per 300 beef carcases and 1 per 1000 sheep or pig carcases and at the corresponding carcase equivalent rate for bulk meat. Bulk meat comprised mainly manufacturing meat (trim) destined for grinding, packed in cartons. Carcase and bulk meat samples are routinely collected under the national monitoring program. Primals comprised cuts individually vacuum packed and chilled. Offals comprised so-called ‘red’ offals such as hearts and livers and ‘green’ offals such as tripes, which were scalded. There is currently no regulatory requirement for the sampling and testing of primal cuts under the national monitoring program. So, for this study, establishments sampled primals and offal at a carcase equivalent rate of 1 per 1000 for beef and 1 per 3000 for sheep and pigs. Primal and offal samples were taken immediately before packing and chilling or freezing, with the exception of one pig establishment which sampled offals after chilling.

Samples were taken by quality assurance personnel at each establishment under the authorisation of the on-plant government inspection service and processed at a laboratory accredited to the ISO/IEC 17025-2005 standard [15] by the National Association of Testing Authorities, Australia. Where the laboratory was located on-site, samples were refrigerated until same-day processing. Samples transported to an off-site laboratory were refrigerated to arrive at 4 °C or cooler and processed the next day.

Bacteria were removed from the carcase by back-and-forth strokes with a single Whirlpak sponge resuscitated in Butterfields solution over an area of 100 cm^2^ at three sites on beef and pig carcases (limit of detection, LOD 0.08 cfu/cm^2^) and 25 cm^2^ at three sites on sheep carcases (LOD 0.33 cfu/cm^2^) [2]. A similar methodology as used for carcases was employed for primals, sponging an area of 100 cm^2^ at a single site on the surface (LOD 0.25 cfu/cm^2^). Excision sampling was used for bulk meat and offal samples, with approximately 25 g, including some outer surface being taken (LOD 10 cfu/g).

### 2.3. Microbiological Analysis

Testing of samples was as per the DAFF-approved methods for the microbiological testing of meat and meat products [16]. For example, bacteria were removed from the sponge either by massaging sponges in a stomacher or by “squishing” sponges by hand in the sample bags for 30 s and, from the moisture expressed, preparing serial dilutions in 0.1% buffered peptone water blanks (9 mL) using 1 mL aliquots. Excision samples were homogenised in a stomacher with 0.1% buffered peptone to give a 10-fold dilution. Aliquots (1 mL) from each dilution were spread on *E. coli* Petrifilm (3M, Sydney, Australia) and Aerobic Plate Count (APC) Petrifilm (3M) and incubated at 30 °C/48 h. Colonies were identified and counted as per the manufacturer’s instructions.

### 2.4. Statistical Analysis

Establishment data were sent to the South Australian Research and Development Institute either daily or weekly for entry into a database. Counts/g or cm^2^ were converted to log_10_ cfu/g or cm^2^ and the statistical analysis (means, analysis of variance and Tukey HSD) was carried out using the statistical software R (version 4.2.2) [17] at a significance level of 0.05.

## 3. Results and Discussion

A total of 27,157 microbiological results were gathered as part of the trial: 15,155 from beef, 8405 from sheep and 3597 from pig establishments comprising 11,512 carcase, 9872 bulk meat, 2169 primal and 3604 offal samples.

**Figure 1:**
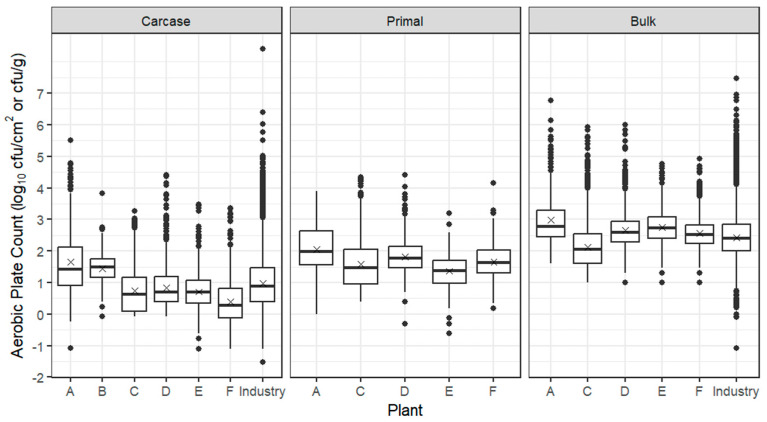
Box plots of the APC of beef carcases (log_10_ cfu/cm^2^), primals (log_10_ cfu/cm^2^) and bulk meat (log_10_ cfu/g) from establishments A–F. The box encompasses data between the 25th and 75th percentiles, with the mean indicated by ‘X’ and median by a solid line. Box plots of the whole industry (ESAM data including trial establishments) are also given for carcases and bulk meat; industry data are currently not collected for primals.

Box plots for APC from beef carcases, primals and bulk meat at individual beef establishments are presented in Figure 1, together with the whole industry combined, based on ESAM data, indicating that the trial establishments were broadly representative of the industry. The mean from carcases from all six establishments was 0.84 log_10_ cfu/cm^2^ with establishment means ranging from 0.39 log_10_ cfu/cm^2^ (establishment F) to 1.65 log_10_ cfu/cm^2^ (establishment A). Two establishments (A and B) had mean APC counts more than 0.5 log_10_ higher than other establishments, which may reflect the fact that these establishments produced carcases slaughtered from long-haired European breeds of *B. taurus*. Industry information indicates that these cattle present challenges during rain events due to build-up of “tag”, a mixture of soil and faeces on hide incision lines, the problem being magnified particularly on feedlot cattle. However, as seen from Table 1, establishment E slaughtered similar stock at a similar line speed in the same geographical region as establishments A and B and produced carcases with much lower APCs. At three northern establishments (C, D and F), the livestock mix contained a substantial proportion of both *B. indicus* and grain-fed cattle, which were slaughtered at line speeds of 100–300 head/hour. The low mean log_10_ APCs on carcases from these establishments are probably linked to slaughter floor interventions: establishments C and D passed carcase sides through a hot water cabinet and at establishment F lactic acid was sprayed on the tail/bung area immediately after stunning. In addition, industry information suggests that short-haired *B. indicus* cattle, particularly those grain-fed for 100 days, are more easily processed on the slaughter floor because the fat layer beneath the hide facilities its removal. However, during the northern raining season, feedlot cattle enter abattoirs with a considerable amount of soil and faecal contamination of the hide, as do European breeds in the southern states. Establishments C and D also differed from other beef establishments by using spray chilling to offset the weight loss, which accompanies air chilling. After overnight chilling, passage of beef carcases through the boning room resulted in higher mean APCs of 0.4–1.3 log_10_ cfu/cm^2^ for primals; bulk meat APCs were also higher, although their comparison with carcases and primals is not possible because counts were obtained by excision sampling (log_10_ cfu/g).

While the prevalence of *E. coli* on beef carcases was generally low, there were more frequent detections at each establishment after fabrication to bulk meat and primals (Table 4). While concentrations remained low on primal meat, higher concentrations were detected from bulk product, possibly because bulk meat has a higher proportion of trim from external carcase surfaces.

With respect to primal cuts, the mean log_10_ APC of primals from the five beef establishments was 1.65 log_10_ cfu/cm^2^ with means for specific primals ranging from 1.41 and 1.42 log_10_ cfu/cm^2^ on internal cuts, such as tenderloins and cube rolls, to 1.80 to 1.99 log_10_ cfu/cm^2^ on cuts with external surfaces such as outside, brisket and blade; not unexpectedly, the prevalence of *E. coli* was also higher on external cuts (Table 5).

**Figure 2:**
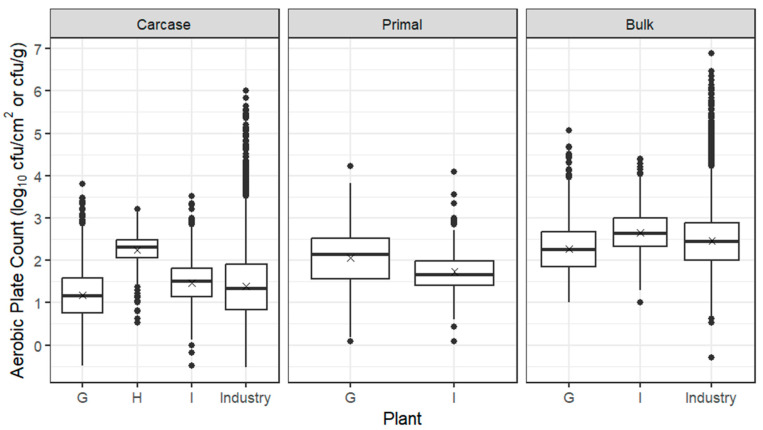
Box plots of the APC of sheep carcases (log_10_ cfu/cm^2^), primals (log_10_ cfu/cm^2^) and bulk meat (log_10_ cfu/g) from Establishments G–I. The box encompasses data between the 25th and 75th percentiles, with the mean indicated by ‘X’ and median by a solid line. Box plots of the whole industry (ESAM data including trial establishments) are also given for carcases and bulk meat; industry data are currently not collected for primals.

Box plots for APC from carcases, primals and bulk meat at individual sheep establishments are presented in Figure 2, together with the whole industry combined for carcases and bulk meat, based on ESAM data, indicating that the trial establishments were broadly representative of the industry. The mean log_10_ APC from carcases across all three sheep establishments was 1.56 log_10_ cfu/cm^2^, with establishment H having a mean around 1 log_10_ units higher than establishments G and I. All three sheep establishments used inverted dressing, but line speed differed from 8/minute (establishment G) to 8.5 (establishment H) and 10 (establishment I), as did the use of a two-knife system and legging paper to prevent roll-back of the pelt and consequent contamination of the forequarters (Table 2).

After overnight chilling and passage of sheep carcases through the boning room, the mean log_10_ APC of primals was around 1 log_10_ cfu/cm^2^ higher at establishment G and less than 0.5 log_10_ cfu/cm^2^ higher at establishment I; bulk meat mean log_10_ APCs at establishments G and I were 2.27 and 2.66 log_10_ cfu/g, respectively. All carcases from establishment H were shipped off-site for fabrication at independent boning rooms, hence the absence of data for primals and bulk meat.

Following boning, the detection of *E. coli* was higher on sheep primals, compared with carcases, with the average concentration of *E. coli* from positive samples of carcases and primals ≤ 0.1 log_10_ cfu/cm^2^ (2 cfu/cm^2^). On excised samples of bulk meat, the prevalence of *E. coli* ranged from 15 to 24%, with the average concentrations ≥ 1.3 log_10_ cfu/g (20 cfu/g) at sheep establishments G and I (Table 6).

The mean log_10_ APC of primals from the two sheep establishments was 1.89 log_10_ cfu/cm^2^ with means for individual primals close to the overall mean. The prevalence of *E. coli* was much higher than on beef primals, especially on legs and shoulders (Table 7).

**Figure 3:**
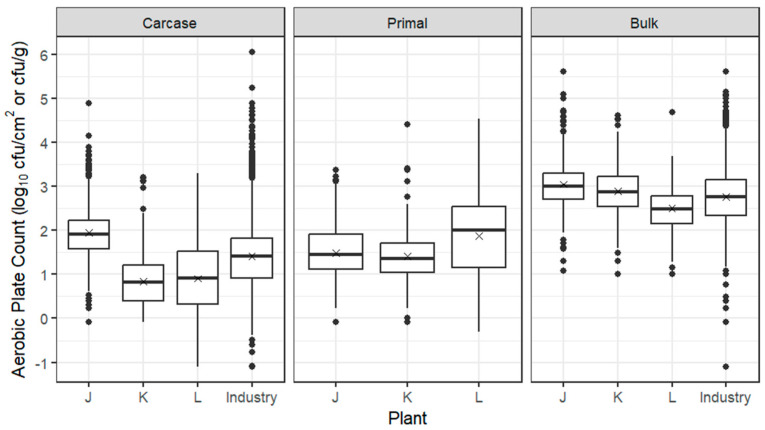
Box plots of the APC of pork carcases (log_10_ cfu/cm^2^), primals (log_10_ cfu/cm^2^) and bulk meat (log_10_ cfu/g) from establishments J–L. The box encompasses data between the 25th and 75th percentiles, with the mean indicated by ‘X’ and median by a solid line. Box plots of the whole industry (ESAM data including trial establishments) are also given for carcases and bulk meat; industry data are currently not collected for primals.

As for beef and sheep, box plots for APC from carcases, primals and bulk meat at individual pork establishments are presented in Figure 3, together with the whole industry combined, based on ESAM data, indicating that the trial establishments were broadly representative of the industry. The mean log_10_ APC from carcases from all three pork establishments was 1.35 log_10_ cfu/cm^2^, with the average being 1 log_10_ cfu/cm^2^ higher at pork establishment J compared with establishments K and L, possibly related to its faster line speed. Steam scalding at establishment K may also be linked with its lower APC. After overnight chilling and passage of pig carcases through the boning room, the mean log_10_ APCs of primals were higher at establishments K and L but lower at establishment J; bulk meat mean APCs ranged between 2.5 and 3.0 log_10_ cfu/g.

Following boning, the prevalence of *E. coli* was lower on pork primals compared with carcases. On excised samples of bulk meat, the prevalence of *E. coli* ranged from 1.4 to 3.9%, with the average concentrations approximately 1.2 log_10_ cfu/g (16 cfu/g) (Table 8).

The mean log_10_ APC of primals from the three pork establishments was 1.57 log_10_ cfu/cm^2^, with means for individual primal cuts similar to the overall mean, with the exception of trotters, which were considerably higher than other primals (Table 9).

Establishments reported APCs for more than 40 offal types, of which the most commonly collected (*n* > 25) are presented in Table 10. All establishments collected ‘red’ offals (hearts, kidneys, livers, etc.) while ‘green’ offals (stomach parts processed by scalding) were collected predominately from beef and sheep. Some offals were specific for only sheep (brains) or pigs (chitterlings, ears, snouts and trotters).

While microbiological quality varied between offal type, there was comparably little variability between the same offals taken from beef, sheep or pig carcases. Offal from organs (heart, liver and kidney) were generally 2 log_10_ cfu/g while tripes were 3 log_10_ cfu/g and tongues were 4 log_10_ cfu/g. It might be expected that organ offals could be removed without significantly increasing their bacterial load, and so would pick up contamination whilst passing down chutes and from handling in the offal room. In contrast, offals derived from the gastrointestinal tract would have a high bacterial loading prior to washing, scalding and cooling, a proportion of which would be retained on the finished product. Tongues and meats derived from the head might also be expected to have a higher bacterial loading, stemming from contamination with saliva. The mean APCs in Table 10 are very similar to those obtained in a contemporaneous survey of chilled and frozen offals from 17 Australian export establishments which stated “the average APC on beef, sheep and lamb offal was 3.25, 3.38 and 3.70 log_10_ cfu/g, respectively” [18].

Previous surveys [4,5,6,7,8,9,10,11] monitored carcases and cuts at establishments which represented approximately 80% of industry output. By contrast, the present 13-month survey monitored establishments representing approximately 26% (beef), 15% (sheep) and 41% (pork) of national output on a daily basis. In addition, the present survey sampled carcases plus products derived from them: primal cuts, bulk meat and offals; for carcases and the derived end products, there was little evidence of seasonal effects on APCs [13].

In Table 11 are presented summary data of surveys of Australian beef and sheep carcases, which all used the same methodology. As a result, it may be construed that there has been a meaningful reduction in total bacterial loadings over the period 1998–2018, reflecting significant improvements in livestock handling, establishment infrastructure, operator training and the uptake of HACCP systems throughout the industry.

Currently, the performance of individual establishments is assessed against criteria set by the Australian regulator, the Department of Agriculture, Fisheries and Forestry (current name) in the *Microbiological Manual for Sampling and Testing of Export Meat and Meat Products* [2], using limits for APC and generic *E. coli* and three-class sampling plans that are assessed on a moving window of consecutive samples (*n* = 15), as described by FAO/WHO [19]. A window failure occurs when the number of marginal results (> m but ≤ M) exceeds c, or a single result exceeds the unacceptable level (M); there are different values for c, m and M according to livestock category [2]. In the present survey, there were 19 failed windows in five establishments over the 13-month survey period—13 for beef, 5 for sheep and 1 for pig carcases (Table 12). No other establishment had a moving window failure.

As set out by DAFF [2], Australia’s current performance monitoring system sets different sampling and evaluation criteria for carcases of bovines, ovines, porcines, caprines, cervines, equines, *Camelidae*, ratites, macropods and wild boars, and for various categories within them (steer/heifers versus cow/bulls). For the three most processed species (bovines, ovines and porcines), the criteria for n, c, m and M were formulated based on performance data from 2001 to 2002 [3]. However, as indicated in Table 11, the hygienic condition of carcases has improved greatly over the ensuing period and the export of carcase parts, particularly primals and offals, has also increased substantially, e.g., offal exports now exceed 200,000 t/annum [20].

The results of this trial have enabled representatives from industry, the regulator and research establishments to develop criteria which better reflect the performance of the current meat industry. Major proposed changes include setting identical criteria for n and c and an m-limit for products (carcases, primals, bulk meat and offals) from all species (beef, sheep, pork, etc.); removing *Salmonella* testing; and reducing frequency of carcase monitoring balanced by monitoring primals, bulk meat and offals. The window system is retained, and failure to meet any criterion for any product triggers an Alert requiring the establishment to review the process to identify any factors that may have caused the Alert and take any corrective and preventative action to control those factors in discussion with the on-plant veterinarian.

The resultant alternative monitoring system is currently being reviewed by Australia’s major trading partners.

## Figures and Tables

**Table 1:** Process characteristics of participating beef establishments.

Characteristic	Establishment					
	A	B	C	D	E	F
*Bos taurus*:*Bos indicus*	100:0	100:0	60:40	50:50	100:0	60:40
Feedlot, grain-fed (%)	1	35	50	48	10	50
Slaughter volume/hour	60	70	300	100	50	125
Two-knife system	No	No	Yes	Yes	Yes	Yes
Separate hide-on/hide-off areas	No	No	Yes	Yes	No	No
Bung sealed	Yes	Yes	Yes	Yes	Yes	Yes
Whole carcase intervention	No	No	Hot water	Hot water	No	No
Chilling	Air (2–4 °C)	Air (2–4 °C)	Spray/Air (2–4 °C)	Spray/Air (2–4 °C)	Air (2–4° C)	Air (2–4 °C)

**Table 2:** Process characteristics of participating sheep establishments.

Characteristic	Establishment		
	G	H	I
Slaughter volume/hour	480	510	600
Inverted dressing	Yes	Yes	Yes
Two-knife system	Yes	No	Yes
Legging paper	Yes	No	No
Bung sealed	Yes	Yes	No
Vacuum cutting lines	Yes	Yes	Yes
Chilling	Air (2–4 °C)	Air (2–4 °C)	Air (2–4 °C)

**Table 3:** Process characteristics of participating pork establishments.

Characteristic	Establishment		
	J	K	L
Slaughter volume/hour	560	260	170
Scalding	Water 60 °C/6 min	Steam 8 min	Water 60 °C/5 min
Bung sealed	No	No	No
Chilling	Air (2–4 °C)	Air (2–4 °C)	Air (2–4 °C)

**Table 4:** Prevalence (%) and (mean log_10_ concentration *) of *E. coli* on carcases (log_10_ cfu/cm^2^), primals (log_10_ cfu/cm^2^) and bulk meat (log_10_ cfu/g) at beef establishments A–F. Mean values with the same letter within the same column are not significantly different.

Establishment	Carcase	Primal	Bulk
A	1.7 (−0.57 ^a^)	6.8 (−0.19 ^a^)	4.5 (1.13 ^a^)
B	2.7 (−0.97 ^a^)	-	-
C	0.7 (−1.06 ^a^)	9.5 (−0.29 ^a^)	1.9 (1.29 ^a^)
D	3.7 (−0.79 ^a^)	7.0 (−0.03 ^a^)	9.2 (1.49 ^a^)
E	5.5 (−0.86 ^a^)	6.5 (−0.17 ^a^)	11.2 (1.30 ^a^)
F	1.1 (−0.89 ^a^)	3.5 (−1.18 ^b^)	4.7 (1.39 ^a^)
Whole of industry	2.7 (−0.64 ^a^)	-	-

* cfu/cm^2^ or cfu/g of detections.

**Table 5:** APC of beef primals (log_10_ cfu/cm^2^) and *E. coli* prevalence (%) at participating beef establishments. Mean values with the same letter are not significantly different.

Primal	n	Mean log_10_ cfu/cm^2^	*E. coli* Prevalence (%)
Tenderloin	105	1.41 ^a^	1.9
Cube roll	106	1.42 ^a^	6.6
Striploin	109	1.43 ^ab^	6.4
Chuck	73	1.52 ^abc^	2.7
Chuck tender	86	1.60 ^abc^	7.0
Eye round	85	1.63 ^abc^	8.2
Rump	94	1.66 ^abcd^	6.4
Navel end brisket	83	1.68 ^abcd^	8.4
Topside	85	1.77 ^bcd^	7.1
Knuckle	116	1.78 ^cd^	8.6
Outside	90	1.80 ^cd^	11.1
Point end brisket	84	1.85 ^cd^	6.0
Blade	94	1.99 ^d^	10.6

**Table 6:** Prevalence (%) and (mean log_10_ concentration *) of *E. coli* on carcases (log_10_ cfu/cm^2^), primals (log_10_ cfu/cm^2^) and bulk meat (log_10_ cfu/g) at sheep establishments G–I. Mean values with the same letter within the same column are not significantly different.

Establishment	Carcase	Primal	Bulk
G	30.9 (0.1 ^a^)	38.4 (−0.06 ^a^)	15.8 (1.30 ^a^)
H	35.3 (−0.07 ^b^)	-	-
I	22.7 (−0.08 ^b^)	28.7 (−0.15 ^a^)	24.0 (1.38 ^a^)
Whole of industry	13.1 (−0.04)	-	-

* cfu/cm^2^ or cfu/g of detections.

**Table 7:** APC of sheep primals (log_10_ cfu/cm^2^) and *E. coli* prevalence (%) at establishments G and I. Mean values with the same letter are not significantly different.

Primal	n	Mean log_10_ cfu/cm^2^	*E. coli* Prevalence (%)
Short loin	39	1.68 ^a^	25.6
Tenderloin	23	1.71 ^a^	26.1
Loin	51	1.78 ^a^	21.6
Leg bone in	66	1.80 ^a^	43.9
Rack	108	1.89 ^a^	16.7
Shoulder bone out	21	1.89 ^a^	23.8
Leg bone out	59	1.91 ^a^	47.5
Shank	27	1.91 ^a^	29.6
Square cut shoulder	79	1.94 ^a^	38.0

**Table 8:** Prevalence (%) and (mean log_10_ concentration *) of *E. coli* on carcases (log_10_ cfu/cm^2^), primals (log_10_ cfu/cm^2^) and bulk meat (log_10_ cfu/g) at pig establishments J–L. Mean values with the same letter within the same column are not significantly different.

Establishment	Carcase	Primal	Bulk
J	5.0 (0.27 ^a^)	3.8 (−1.07 ^a^)	3.9 (1.21 ^a^)
K	5.9 (−0.09 ^b^)	3.4 (−0.92 ^a^)	1.4 (1.23 ^a^)
L	5.4 (−0.02 ^c^)	2.1 (1.00 ^b^)	-
Whole of industry	3.8 (−0.50)	-	-

* cfu/cm^2^ or cfu/g of detections.

**Table 9:** APC of pork primals (log_10_ cfu/cm^2^) at establishments J–L. Mean values with the same letter are not significantly different.

Primal	n	Mean log_10_ cfu/cm^2^
Tenderloin	32	1.32 ^a^
Ribs	33	1.34 ^a^
Belly	40	1.40 ^a^
Loin	28	1.44 ^a^
Middle	24	1.49 ^a^
Leg	36	1.55 ^a^
Shoulder	32	1.55 ^a^
Topside	20	1.61 ^ab^
Collar butt	25	1.66 ^ab^
Trotter	32	2.38 ^b^

**Table 10:** Mean log_10_ APC (log_10_ cfu/g) of beef, sheep and pig offals. Mean values with the same letter within the same column are not significantly different.

Offal	Beef		Sheep	Pig		
	n	Mean	n	Mean	n	Mean
Aorta	29	1.82 ^a^	-	-	-	-
Diaphragm	27	1.95 ^a^	-	-	-	-
Tendon	28	2.60 ^abc^	-	-	-	-
Omasum	113	2.79 ^b^	-	-	-	-
Liver	146	2.84 ^b^	230	2.96 ^a^	38	2.37 ^a^
Kidney	100	2.90 ^bc^	258	2.90 ^a^	38	2.50 ^a^
Honeycomb	135	2.92 ^bc^	-	-	-	-
Heart	143	2.94 ^bc^	239	2.79 ^a^	30	2.26 ^a^
Tripe pieces	104	3.15 ^bcde^	307	3.69 ^c^	-	-
Skirt	285	3.18 ^cd^	84	3.33 ^b^	-	-
Tail	121	3.56 ^ef^	29	3.64 ^bc^	-	-
Mountain chain	84	3.57 ^defg^	-	-	-	-
Head meat	241	3.74 ^fg^	-	-	-	-
Tongue	209	3.98 ^g^	45	4.48 ^d^	60	4.49 ^c^
Brain	-	-	30	3.58 ^bc^		
Trotter	-	-	-	-	31	3.61 ^b^
Snout	-	-	-	-	33	3.78 ^b^
Ear	-	-	-	-	36	3.90 ^b^
Chitterling	-	-	-	-	26	4.59 ^c^
All combined		3.23		3.18		3.37

**Table 11:** Beef and sheep carcase contamination in Australia from 1998 to 2018.

	n	Mean APC (log_10_ cfu/cm^2^)	Reference
Beef *			
1998	1268	2.4	[6]
2004	1147	1.3	[8]
2018	6016	0.8	Present study
Sheep **			
1998	917	3.5	[7]
2004	1117	2.3	[9]
2018	3693	1.6	Present study

* LOD 0.08 cfu/cm^2^. ** LOD 0.33 cfu/cm^2.^

**Table 12:** Failed windows for APC and *E. coli* on beef, sheep and pig carcases; in the ‘m’ column are listed the number of failures due to exceeding ‘m’ too many times in the moving window, while in the ‘M’ column are listed the number of failures due to exceeding ‘M’.

Establishment	APC Failed Windows		*E. coli* Failed Windows	
	m	M	m	M
A	6	5	0	0
D	0	0	1	0
E	0	0	1	0
G	0	0	0	5
J	0	1	0	0

## Data Availability

The data presented in this study are available on request from the corresponding author. The data are not publicly available due to privacy obligations to the participating processing establishments.
